# Relationship between intrinsic foot muscle weakness and pain: a systematic review

**DOI:** 10.1186/1757-1146-7-S1-A51

**Published:** 2014-04-08

**Authors:** Penelope J Latey, Joshua Burns, Claire Hiller, Elizabeth J Nightingale

**Affiliations:** 1 Arthritis and Musculoskeletal Research Group, Faculty of Health Sciences, University of Sydney, NSW, Australia; 2 Institute for Neuroscience and Muscular Research, The Children's Hospital at Westmead, Sydney, NSW, Australia

## Background

Foot muscle weakness has been linked with painful foot pathologies. This systematic review evaluated the relationship between foot muscle weakness and foot pain in adults.

## Methods

Electronic databases (AgeLine, MEDLINE, CINHAL, AMED, Scopus, SPORT Discus, Web of Science) and reference lists were searched for all years up to March 2013. Two independent reviewers rated all included papers for methodological quality using a modified checklist from the Quality Index Tool. Due to the heterogeneity of studies, no data were pooled for meta-analysis.

## Results

Seven studies evaluated the relationship between foot muscle weakness and foot pain. Methodological quality varied from poor (40%) to very good (89%). Four studies reported a significant relationship between foot muscle weakness and foot pain. Participants with plantar fasciitis were reported to have significant foot pain associated with a decrease in the cross-sectional area of the forefoot musculature and reduced toe flexor force. A study considering non-specific foot pain found a significant difference in dynamic toe flexor force between participants with disabling foot pain versus no pain on some day(s) and on most/every day. Finally, a clinical trial evaluating hallux limitus reported a significant improvement in pain and hallux plantar muscle strength after treatment. Of the three studies reporting no association, two reported only on hind foot muscles and one had a restricted sample. Summary of data extracted and quality index scores is shown in Table 1.

**Table 1:** Summary of Participant Characteristics, Outcome Measures and Quality Index Scores of Included Studies

	**Munteanu *et al.* 2012**[1]	**Duranti *et al.* 1985**[2]	**Schmid *et al.* 2009**[3]	**Chang *et al.* 2012**[4]	**Allen *et al.* 2003**[5]	**Mickle *et al.* 2011**[6]	**Shamus *et al.* 2004**[7]
**Sample size**	n=151	A: n=15	A: n=80	A: n=8	A: n=20	n=312	A: n=10

		B: n=5	B: n=80	B: n=8 Bilat	B: n=20		B: n=10

**Mean age (SD), yrs**	54.5 (11.2)	A: 55.4	A: 48	44.9 (8.4)	A: 44.9 (9.2)	71 (6.5)	A: 32 (6.3)

		B: 51.7	B: 48		B: 43.1 (8.0)		B: 33.6 (5.4)

**Gender**	95 M 56W	A: 5M 10W	A: 38M 42W	1M 7 W	A: 4M 16W	158M 154W	A: 2M 8W

		B: 2M 3W	B: 38M 42W		B: 4M 16W		B: 3M 7W

**Pathology^a^**	OA of 1^st^ MPJ	HV/Chronic pain	Foot pain	Plantar fasciitis	Plantar fasciitis	Foot pain	Hallux limitus

**Muscle tests^b^**	Direct/PP	Indirect/EMG	Indirect/MRI	Indirect/MRI	Direct/St. G	Direct/PP	Direct/Dyno

**Pain scales^c^**	FHSQ	P/A	P/A	FFI	P+≥ 2 mths/A	MFPDI	Verbal p scale

**Association**	No	No	No	No/Yes	Yes	Yes	Yes

**Quality Index Score**	81%	40%	56%	82%	78%	89%	71%

Legend: A: Symptomatic Group B: Control Group Bilat - bilateral feet as control.

Pathology^a^: OA of 1^st^ MPJ- osteoarthritis of 1^st^ metatarsophalangeal joint; HV- hallux valgus.

Muscle tests^b^: Indirect: MRI- magnetic resonances imaging, EMG- electromyography. Direct: PP- pressure plate, St. G-strain gauge, Dyno- dynamometry.

Pain scales^c^: FHSQ- Foot health survey questionnaire; P/A- present or absent; P + ≥ 2mths- present plus greater than or equal to 2 months duration;

FFI- Foot function index; MFPDI-Manchester foot pain and disability index; Verbal pain scale

## Conclusion

Despite some conflicting data encountered in this systematic review, there is evidence of a significant association between foot pain and muscle weakness, primarily related to toe flexion and foot pain, when the pain is of frequent disabling intensity.
